# Parental education related to their children’s health in late childhood and early adolescence for Pacific families within New Zealand

**DOI:** 10.1038/s41598-022-09282-x

**Published:** 2022-03-29

**Authors:** Philip J. Schluter, Jesse Kokaua, El-Shadan Tautolo, Leon Iusitini, Rosalina Richards, Troy Ruhe

**Affiliations:** 1 School of Health Sciences – Te Kura Mātai Hauora, and Child Well-being Research Institute – Te Kāhui Pā Harakeke, University of Canterbury – Te Whare Wānanga o Waitaha, Private Bag 4800, Christchurch, 8140 New Zealand; 2 School of Clinical Medicine, Primary Care Clinical Unit, The University of Queensland, Brisbane, Australia; 3 Division of Health Sciences, Va’a O Tautai, University of Otago, Dunedin, New Zealand; 4 Centre for Pacific Health and Development Research, Auckland University of Technology, Auckland, New Zealand

**Keywords:** Diseases, Risk factors

## Abstract

Pacific people continue to carry a disproportionately heavy social and health burden relative to their non-Pacific peers in New Zealand, and those with less formal education are experiencing social and health declines. Improving education and educational needs is seen as being central to decreasing these health inequities. While expansive, the empirical evidence-base supporting this stance is relatively weak and increasingly conflicting. Using a large birth cohort of 1,368 eligible Pacific children, together with their mothers and fathers, this study longitudinally investigates the relationship between paternal education levels and sentinel measures of their children’s physical health, mental health and health risk taking behaviours during late childhood and early adolescence. In adjusted analyses, it was found that mothers and fathers who undertook further schooling over the 0–6 years postpartum period had children with significantly lower logarithmically transformed body mass index increases at 11-years and 14-years measurement waves compared to 9-years levels than those who did not study (p = 0.017 and p = 0.022, respectively). Furthermore, fathers who undertook further schooling over this 0–6 years postpartum period also had children with significantly lower odds of risk taking behaviours (p = 0.013). These results support policy aimed at increasing educational opportunities for Pacific people in New Zealand.

## Introduction

Education and health are inextricably yet complexly linked^1^. Exposure to formal education is often referred to as a leading “social vaccine”; a social intervention that provides resources that can protect individuals and improve the health of populations^1,2^. Economically, it has been asserted that policies which increase rates of completion of high school and college degrees could result in longer, healthier lives and substantial economic value for the population^3^. While a burgeoning number of studies demonstrate that increased educational attainment is generally associated with better health across many contexts and outcomes^4^, the establishment of a causal link has been more challenging^5^. Moreover, at times, null or apparently contradictory relationships emerge^1,4^. Many questions remain; as has the call for more research at this critical time when health differences are widening and those with less formal education are experiencing social and health declines^4^.

A multiplicity of studies also show that higher parental education, particularly maternal education, is associated with health success in their children^6–9^. Although parental educational hypergamy (i.e., education heterogeneity between parents) may be detrimental to child health success^10^, and some have asserted that the parental education effect is weakening—at least in low and middle income countries^11^. Numerous hypothesised pathways explaining this parental educational advantage have been proposed, each supporting a wide range of preventive and treatment oriented behaviours attributed directly to proximate determinants of child health^8,11^. However, much of this evidence-base is premised on observational studies, or natural experiments which have exploited education policy reforms. These results are subject to potential confounding bias due to germane unobserved variables (such as: income, innate ability and other endowments) associated with both education and child health^11^. Moreover, this research typically regards parental education as a ‘static’ time-invariant factor, and health policy recommendations thus invariably target cohorts of future parents rather than existing parents. Additionally, most research has investigated mothers’ impacts, rather that fathers’, as women have historically been the primary caregivers. Despite fathering role and behaviour changes in many contemporary societies, their importance and impact on child development have often been overlooked. This has lead bodies such as World Health Organization^12^, and the United Nations Secretariat^13^, to call for father-specific investigations and a deeper understanding. We could find no population-base studies which specifically investigate the acquisition of further postpartum maternal or paternal education and improved health outcomes in their children.

While many Pacific communities in New Zealand are thriving, Pacific people continue to carry a disproportionately heavy social and health burden relative to their non-Pacific peers^14,15^. From the most recent 2018 Population Census, 381,642 (8.1%) Pacific people were usually resident in New Zealand, with median age 23.4 years (compared to 41.4 years for New Zealand European [NZE] people, the dominant ethnic group comprising 70.2% of the total population), 66.4% were born in New Zealand (compared to 82.8% of NZE people), with median individual adult income of $24,300 (compared to $34,600 for NZE people), and 24.5% had no secondary school or tertiary educational qualification (compared to 18.2% for NZE people)^16^. The Pacific population is also becoming increasingly diverse; with many identifying with two or more Pacific and/or non-Pacific ethnicities, and having both ancestral Pacific Island homelands and contemporary New Zealand values and cultural practices^14,15^. Despite this diversity, enduring culturally-specific values are commonly shared among Pacific people, which include the importance of family, collectivism and communitarianism, spirituality, reciprocity and respect^15^.

The Ministry of Health’s Ola Manuia Pacific Health and Wellbeing Action Plan 2020–2025 targets better, fairer and more equitable health outcomes for Pacific people^15^. Consistent with the social determinants of health framework^17^, improving education is seen within the Action Plan as one of the primary factors needed for improving Pacific peoples’ health. Strategies which understand and improve education and educational needs are seen as being central to decreasing health inequities and providing healthier living and working environments for Pacific communities. The Action Plan also recommends the use of Pacific data to drive evidence-based actions that improve Pacific outcomes across a variety of domains that extend beyond a sole health focus^15^. One Pacific derived framework which enables this is the Fonofale model^18^; whereby a Samoan fono (meeting) fale (house) is used as a pictorial analogy for health. The fale’s roof is comprised of cultural values and beliefs, seen as a shelter for life, while the floor or foundation represents family. Connecting culture and family are four inter-related pou (pillars) signifying physical, mental, spiritual, and other dimensions. The fale itself rests in a cocoon of three final factors: environment, time, and context.

When considering the population burdens disproportionately carried by Pacific people within New Zealand, obesity (physical pou), poor youth mental health (mental pou), and risk taking behaviours (other pou) stand out^19^. Motivated by these heavy and unequally shared burdens, the Acton Plan and Ministry of Health priority areas, the call for Pacific-specific research, the increasingly conflicting results associated with parental education and child health, and the lack of population-based studies investigating the effect of parental educational acquisition, we seek to provide apposite empirical evidence. Using a birth cohort study, which includes the mother-father-child triad, this study aims to investigate the relationship between highest maternal and paternal education levels prior to their child’s birth, the further education engagement or qualification acquisition within the post-delivery period, and sentinel physical health, mental health and child risk behaviour measures of health in Pacific children during late childhood and early adolescence (ages 9–14 years).

## Methods

### Study design

The Pacific Islands Families (PIF) Study longitudinally follows a cohort of 1,398 Pacific infants born at Middlemore Hospital, South Auckland, New Zealand between 15 March and 17 December 2000.

### Participants

Participants include the mother-father-child triad. All potential participants were selected from births where at least one parent was identified as being of a Pacific ethnicity and a New Zealand permanent resident. Recruitment was made through the Hospital’s Birthing Unit, and consent was sought to make a home visit. For the purpose of this study, only infants’ birth mothers and fathers, singleton infants, and first-born from multiple live births were included.

### Procedure

Approximately 6-weeks postpartum, potential maternal participants were visited at home by female Pacific interviewers fluent in both English and Pacific language(s). Once eligibility was confirmed and written informed consent obtained, mothers participated in approximately 1-h interviews. With written informed consent, home visits were repeated approximately 1 year, 2 years, 4 years, 6 years, 9 years, 11 years and 14 years postpartum. At the 1-year, 2-years, 6-years, and 11-years interviews, mothers were asked to give permission for a male Pacific interviewer to contact and interview the child’s father. With this permission, and informed paternal consent, interviews with fathers were then conducted. At the 14-years measurement wave, mothers were asked to give permission for the child’s father to be mailed a postal survey. With permission, this was then conducted. Moreover, at the 9-years, 11-years and 14-years interviewers, mother were also asked to give their consent for an interviewer to contact and interview their child. This interview was normally conducted at the child’s school and was undertaken only after receiving parental consent and written informed child assent. Detailed information about the cohort, utilised instruments, and procedures is described elsewhere^20,21^.

### Primary childhood outcome measures

Three Fonofale model pou (physical health, mental health, and other) elicited over the 9-years, 11-years and 14-years measurement waves were considered, taking sentinel measures in each. For physical health, body mass index (BMI) was utilized, derived from weight and height (kg/m^2^), and categorised according to the World Health Organization BMI growth reference classification standardised for age (in months) and sex^22^. Underweight was defined as having a z-score < − 2; normal as having − 2 ≤ z-score ≤ 1; overweight as having 1 < z-score ≤ 2; and, obese as z-score > 2. For mental health, the 10-item Children’s Depression Inventory short form (CDI:S) was employed^23,24^. The CDI:S is a self-rated symptom-orientated screen for depressive symptoms in children aged 7 to 17 years. It has robust internal consistency (Cronbach's α = 0.84), reliability, and adequate validity^25,26^. The CDI:S responses fall on a 0–2 Likert scale, and then are summed (five items are reversed scored) to form an overall score which can range from 0 to 20. A higher CDI:S score represents a greater level of depression. For child risk behaviours, a composite score was developed and employed. In the 9-years and 11-years assessments, children were asked whether they has tried: (1) alcohol; (2) cigarettes; (3) drugs (e.g., marijuana, other)—each having response options: Yes, No. Child risk behaviour was indicated in there was an affirmative response to any of these questions. For the 14-years assessment, children were asked on how many days during the past month did they: (1) smoke cigarettes; (2) drink alcohol; (3) use marijuana; (4) use an illegal drug (not counting cigarettes, alcohol or marijuana)—each having response options: never, 1–2 days, 3–5 days, 6–9 days, and 10 or more days. Child risk behaviour was indicated if there was any declared use to any of these questions on alcohol, cigarettes and drug use.

### Primary parental education measures

These were derived from questions on participants’ highest education qualification and current occupation. For mothers, *highest education qualification at baseline* (at the 6-weeks wave) was elicited, and grouped into no formal qualification, secondary school, or post-secondary (which includes: trade or technical certificates; polytechnic or university certificates, diplomas, or degrees). This same question was elicited over the 1-year, 2-years, 4-years, and 6-years measurement waves. *Further study (over 0–6 years)* was indicated if there was an increase in highest education qualification in 1-year, 2-years, 4-years, or 6-years wave compared to baseline or if the mother stated they were a student in any of the 6-weeks to 6-years measurement waves. *Student currently* was treated as being time varying at each measurement wave (9-years, 11-years, and 14 years) and indicated (with ‘1’) if the mother stated they were a student at that measurement wave (otherwise it was set to ‘0’). For fathers, the same definitions were employed except *highest education qualification at baseline* was elicited at the 1-year wave, *further study (over 0–6 years)* used 2-years and 6-years responses, while *student currently* was based on 11-years and 14-years information.

### Sociodemographic and potentially confounding variables

Sociodemographic variables measured at baseline included parents’ age, ethnicity, years lived in New Zealand, relationship status, cultural orientation, household income, and family size. Here, ethnicity was self-identified and participants were classified as Samoan, Tongan, Cook Islands Māori, other Pacific (which includes parents identifying equally with two or more ethnic groups), and non-Pacific (eligible for inclusion through the Pacific ethnicity of the other parent)^20^. Cultural orientation was conceived and measured using Berry’s bi-directional acculturation modelling framework^27^, and orientation toward New Zealand ‘mainstream’ (dominant) culture and Pacific culture measured separately via an adaption of the General Ethnicity Questionnaire^28^. This adapted instrument exhibited acceptable internal consistency (Cronbach’s α = 0.81 and 0.83 for New Zealand and Pacific scales, respectively)^29^. Depending on the individual score falling below or above the group median on each scale, cultural orientation was assigned as: integrator (higher Pacific/higher New Zealand orientation); separator (higher Pacific/lower New Zealand orientation); assimilator (lower Pacific/higher New Zealand orientation); or marginalist (lower Pacific/lower New Zealand orientation).

### Statistical analysis

Reporting of analyses were informed by the STROBE guidelines^30^. All analyses were performed using Stata SE version 17.0 (StataCorp, College Station, TX, USA), and two-sided α = 0.05 defined statistical significance. Participant recruitment and retention numbers were initially reported, followed by baseline descriptive characteristics of mothers and fathers. Child outcome data over the 9-years, 11-years and 14-years measurement waves were then explored and described. The empirical distribution of BMI values was skewed so, as employed elsewhere, a logarithmic transformation used to make them approximately normal^31^. Also as undertaken previously^32^, missing values for individual items for the CDI:S were imputed for up to five items per individual/time point using person-median substitution, where missing values were estimated based on the median for every person at that particular time point^33^. Those with six or more items missing were not imputed, and their CDI:S scores were set to missing. While these discrete scores are restricted to the 0–20 ranges, the data were over-dispersed (with variance/mean ratio > 1), and thus treated as being negative binomial in distribution. Child risk behaviours were binary, and modelled using a binomial likelihood function. Apposite generalized estimating equation (GEE) models, with default canonical link functions, unstructured correlation matrices and robust Huber-White sandwich variance estimators, were then used to separately model the child health indicators, partitioned by mothers and fathers. When a normal likelihood distribution was assumed, then $\widehat{\beta }$ regression coefficients were reported; when a binomial likelihood was assumed, then odds ratios (ORs) were reported, and when a negative binomial likelihood distribution was used, then incidence rate ratios (IRR) were given. In all GEE analyses, both highest education qualification at baseline and further study (over 0–6 years) were treated as time invariant, while being a current student at measurement waves 9-years, 11-years and 14-years was treated as being time varying. Indicator years were used for measurement wave, rather than as a numeric variable, to accommodate potential non-linear patterns and the different definition in child risk behaviour at 14-years compared to the 9-years and 11-years characterisations. As father’s completed 11-years and 14-years interviews, but not the 9-years wave, only child data for the 11-years and 14-years were used in their analyses. Finally, two models for each parent was considered: (1) a crude analysis, that included the parental education and measurement wave variables only; and (2), an adjusted analysis that additionally included the available sociodemographic and potentially confounding variables. Pattern of attrition over time were evaluated using binomial GEE models. In these analyses mothers and fathers were coded with ‘0’ if present and ‘1’ if missing at each measurement wave. Statistical significance of variables included with the GEEs was assessed via Wald’s Type III score statistic. However, in the spirit of Sun and colleagues, no variable selection was made in these adjusted analyses^34^.

### Ethics

Ethical clearance was obtained from the Auckland Branch of the National Ethics Committee X (6-weeks [baseline] and 2-years measurement waves; reference number 99/055), the Northern X Ethics Committee (4-years and 6-years measurement waves; reference number AKY/04/02/019), the Northern Y Regional Ethics Committee (9-years and 11-years measurement waves; reference number NTY/08/12/119), and the Southern Health and Disability Ethics Committee (14-years measurement wave; reference number 13/STH/159). Conduct of the study complied with the ethical standards for human experimentation as established by the Helsinki Declaration. All methods were performed in accordance with that Ethics Committees’ relevant guidelines and regulations. In addition to obtaining maternal and paternal written informal consent before their participation at every measurement wave, at each of the 9-years, 11-years, and 14-years measurement waves informed consent was also obtained from mothers for the study’s child interviewers to contact their children to invite them to participate. Only those receiving this maternal consent were contacted. Informed written assent was then obtained from the children. Participants were free to not participate or withdraw at any time without penalty. The study only included those parents who provided written informed consent and children who provided written informed assent to their data being used.

## Results

### Participants

Overall, 1,708 mothers were identified, 1,657 were invited to participate, 1,590 consented to a home visit and 1,477 were found to be eligible for the PIF Study. Of these, 1,376 (93.2%) mothers participated at the baseline 6-weeks interview of whom 1,368 were birth mothers (6 were adoptive mothers, 1 was a foster mother, and 1 was another interviewee) and included within this study. At the 1-year interview, 825 fathers participated of whom 821 were biological fathers (4 were adoptive fathers) and included here. Of the eligible 1,368 singleton or first-born from multiple births, assessment data were available for 874 (63.9%) children at 9-years, 935 (68.3%) at 11-years, and 916 (67.0%) at 14-years. Overall, 699 (64.4%) children participated in all three measurement waves, 242 (22.3%) participated in two, and 144 (13.3%) participated in a single measurement wave. Thus 1,085 (79.3%) of the eligible 1,368 children had at least one included assessment. Figure S1 in the supplementary materials includes the participant numbers for each measurement wave included within this study.

### Sociodemographics

Baseline demographics of mothers (at the 6-weeks measurement wave) and fathers (at the 1-year measurement wave) are given in Table 1. Most were of Samoan ethnicity, born overseas but living in New Zealand for ≥ 10 years, partnered, living in a household of 5–7 people, and having household income of $20,001-$40,000. By comparison, the year 2000 median annual household income for those residing within the Auckland region was NZ$47,892^35^.

**Table 1:** Baseline sociodemographic characteristics of mother (6-weeks measurement wave, N = 1,368) and father (12-months measurement wave, N = 821) participants.

	Mothers	Fathers
	n (%)	n (%)
**Age (years)**^**a**^		
< 20	110 (8.0)	7 (0.9)
20–29	720 (52.6)	312 (38.1)
30–39	498 (36.4)	390 (47.6)
≥ 40	40 (2.9)	110 (13.4)
**Ethnicity**		
Samoan	647 (47.3)	440 (53.6)
Tongan	287 (21.0)	199 (24.2)
Cook Islands Maori	229 (16.7)	72 (8.8)
Other Pacific^b^	106 (7.7)	54 (6.6)
Non-Pacific	99 (7.2)	56 (9.8)
**Highest education qualification at baseline**^**a**^		
No formal qualification	533 (39.0)	477 (58.2)
Secondary	460 (33.6)	220 (26.9)
Post-secondary	375 (27.4)	122 (14.9)
**Further study (over 0–6 years)**^**a**^		
No	915 (66.9)	724 (88.4)
Yes	453 (33.1)	95 (11.6)
**Years lived in New Zealand**^**c**^		
0–4	213 (15.6)	101 (12.3)
5–9	162 (11.9)	177 (21.6)
≥ 10 and born elsewhere	552 (40.4)	346 (42.1)
≥ 10 and New Zealand born	438 (32.1)	197 (24.0)
**Relationship status**		
Married or de facto	1,100 (80.4)	785 (95.6)
Single	268 (19.6)	36 (4.4)
**Cultural orientation**^d^		
Integrator (higher Pacific/higher NZ)	233 (17.2)	116 (14.1)
Separator (higher Pacific/lower NZ)	443 (32.6)	313 (38.1)
Assimilator (lower Pacific/higher NZ)	434 (32.0)	298 (36.3)
Marginalist (lower Pacific/lower NZ)	247 (18.2)	94 (11.4)
**Household income (NZD)**		
≤ $20,000	454 (33.2)	
$20,001-$40,000	708 (51.8)	
> $40,000	159 (11.6)	
Unknown	47 (3.4)	
**Family size**^**e**^		
2–4	282 (20.6)	
5–7	689 (50.4)	
≥ 8	396 (29.0)	

^a^2 (0.2%) values missing for fathers; ^b^includes those identifying equally with two or more Pacific Island groups; ^c^3 (0.2%) values missing for mothers; ^d^11 (0.8%) values missing for mothers; ^e^1 (0.1%) values missing.

At the 9-years, 11-years and 14-years measurement waves, respectively, 438 (50.1%), 470 (50.3%), and 449 (49.0%) children were female, and the median age at interview was 9.4 years (Q_1_ = 9.2, Q_3_ = 9.8 years), 11.1 years (Q_1_ = 11.0, 11.2 years), and 14.3 years (Q_1_ = 14.0, Q_3_ = 14.6 years).

### Parental education

Table 1 also includes mothers’ and fathers’ highest education qualification at baseline, together with a variable that captures within six years postpartum whether further qualifications were obtained or participants ever identified as being a student. Most participants had no formal qualification at baseline nor enrolled in further study, although relatively more mothers self-reported having post-secondary qualifications at baseline and were undertaking further study compared to fathers (27.4% vs. 14.9% and 33.1% vs. 11.6%, respectively). A similar pattern was observed during the subsequent measurement waves. At 9-years, 11-years and 14-years waves, respectively, 32 (3.5%; from n = 907 valid responses), 47 (4.9%; from n = 959), and 45 (5.1%; from n = 885) mothers identified as being a student, while at waves 11-years and 14-years, respectively, 12 (1.7%; from n = 703) and 6 (1.0%; from n = 625) fathers made the same student identification.

### Child outcomes

Summary descriptive statistics of the child outcome data, by measurement wave, is presented in Table 2; and distributions for logarithmically transformed BMI scores [henceforth ln(BMI)] and CDI:S scores appear in Figures S2 and S3 within the supplementary materials. Although ln(BMI) was analysed within the GEE models, age and sex adjusted BMI categories were also presented to aid interpretation. Using these categories, many children were classified as being obese, with over half of the participants indicated at the 14-years measurement wave. Conversely, a decreasing number were classified have having a normal BMI; with 21.3% having this classification at the 14-years wave.

**Table 2:** Description of the child outcome data, by measurement waves.

	Measurement waves					
	9-years^a^		11-years^b^		14-years^c^	
	n	(%)	n	(%)	n	(%)
**BMI categories**						
Underweight	4	(0.5)	16	(1.7)	4	(0.4)
Normal	216	(24.9)	221	(23.7)	191	(21.3)
Overweight	240	(27.7)	267	(28.6)	247	(27.5)
Obese	407	(46.9)	429	(46.0)	456	(50.8)
**Child’s risk behaviours: smoking, alcohol, drugs**						
No	829	(95.2)	877	(94.1)	792	(89.7)
Yes	42	(4.8)	55	(5.9)	91	(10.3)

	Median	(Q_1_, Q_3_)	Median	(Q_1_, Q_3_)	Median	(Q_1_, Q_3_)
ln(BMI)	3.06	(2.91, 3.23)	3.13	(2.98, 3.28)	3.30	(3.13, 3.47)
CDI:S score^d^	3	(1, 4)	1	(0, 3)	1	(0, 2)

^a^BMI missing for 7 (0.8%), risk behaviours missing for 3 (0.3%), and CDI:S missing for 4 (0.5%) children; ^b^BMI missing for 2 (0.2%), risk behaviours missing for 3 (0.3%), and CDI:S missing for 2 (0.2%) children; ^c^BMI missing for 18 (2.0%), risk behaviours missing for 33 (3.6%), and CDI:S missing for 59 (6.4%) children; ^d^CDI:S scores range from 0 to 20, with higher scores representing a greater level of depression.

In terms of risk behaviours, at the 9-years measurement wave 30 (3.4%) children reported ever consuming alcohol, 16 (1.8%) had tried cigarettes, and none had tried drugs. By the 11-years wave, ever tried alcohol numbers increased to 40 (4.3%) children, cigarettes to 25 (2.7%), and none reported any drug taking. At the 14-years wave, in the past month, 56 (6.3%) children reported drinking alcohol, 58 (6.5%) had smoked cigarettes, 32 (3.6%) used marijuana, and 17 (1.9%) children reported other illegal drug use.

Most of the CDI:S scores had valid responses, without requiring imputation. At the 9-years wave, 10 (1.1%) children had one question imputed and 1 (0.1%) child had four; at the 11-years wave, 28 (3.0%) children had one question imputed, 16 (1.7%) had two, 3 (0.3%) had three, 4 (0.3%) had four, and 1 (0.1%) had five; however, at the 14-years wave, 179 (19.5%) children had one question imputed, 140 (15.3%) had two, 108 (11.8%) had three, 65 (7.1%) had four, and 32 (3.5%) had five questions requiring imputation. In addition to the Table 2 descriptions, CDI:S scores ranged from 0 (n = 157; 18.0%) to 14 (n = 2; 0.2%) at the 9-years wave, from 0 (n = 292; 31.2%) to 12 (n = 3; 0.3%) at the 11-years wave, and from 0 (n = 382; 41.7%) to 18 (n = 1; 0.1%) at the 14-years wave.

### GEE analyses

Crude and adjusted GEE regression estimates and associated 95% CIs for child outcomes at measurement waves 9-years, 11-years, and 14-years for mothers and fathers are presented in Table 3. In the analyses of children’s ln(BMI), a significant increase by measurement wave was observed in all analyses (all p < 0.001). Mothers’ baseline highest education level or being a current student when their child’s outcomes were measured was not associated with ln(BMI) in crude (p = 0.66 and p = 0.71) or adjusted (p = 0.31 and p = 0.62) analyses. However, mothers who undertook further schooling over the 0–6 years postpartum period had children with significantly lower ln(BMI) increases at 11-years and 14-years measurement waves compared to 9-years levels than those who did not study (crude p = 0.016 and adjusted p = 0.017). Similar findings were observed among fathers. After accounting for the significant year effect, being a current student was not associated with children’s ln(BMI) (crude p = 0.25 and adjusted p = 0.25). While baseline highest education level was significant in the crude analysis (p = 0.018), with children of fathers with no formal qualifications having higher ln(BMI) than children with fathers with post-secondary qualifications, this association lost significance in the adjusted analyses (p = 0.58). However, fathers who undertook further schooling over the 0–6 years postpartum period had children with significantly lower ln(BMI) increases at 11-years and 14-years measurement waves compared to 9-years levels than those who did not study (crude p = 0.008 and adjusted p = 0.022).

**Table 3:** Crude and adjusted GEE regression estimates and associated 95% CIs for child outcomes at measurement waves 9-years, 11-years, and 14-years.

Child ln(BMI)		Mothers					Fathers			
		$\widehat{\beta }$(95% CI)		p	Adjust. $\widehat{\beta }$ (95% CI)	P	$\widehat{\beta }$(95% CI)	p	Adjust. $\widehat{\beta }$ (95% CI)	p
**Measurement wave**				< 0.001		< 0.001		< 0.001		< 0.001
9-years		0 (reference)			0 (reference)					
11-years		0.062 (0.054, 0.070)			0.062 (0.054, 0.070)		0 (reference)		0 (reference)	
14-years		0.236 (0.228, 0.245)			0.236 (0.228, 0.245)		0.182 (0.171, 0.193)		0.182 (0.171, 0.193)	
**Highest education qualification at baseline**				0.66		0.31		0.018		0.58
No formal qual		−0.012 (−0.043, 0.020)			−0.025 (−0.059, 0.010)		0.064 (0.016, 0.112)		0.028 (−0.024, 0.079)	
Secondary school		0.002 (−0.031, 0.034)			−0.005 (−0.038, 0.029)		0.029 (−0.025, 0.083)		0.016 (−0.038, 0.069)	
Post-secondary school		0 (reference)			0 (reference)		0 (reference)		0 (reference)	
**Further study (over 0–6 years)**				0.016		0.017		0.008		0.022
Yes		−0.033 (−0.059, −0.006)			−0.033 (−0.060, −0.006)		−0.063 (−0.110, −0.017)		−0.056 (−0.104, −0.008)	
No		0 (reference)			0 (reference)		0 (reference)		0 (reference)	
**Student currently**				0.71		0.62		0.25		0.25
Yes		0.004 (−0.019, 0.027)			0.006 (−0.017, 0.029)		−0.057 (−0.153, 0.040)		−0.056 (−0.152, 0.039)	
No		0 (reference)			0 (reference)		0 (reference)		0 (reference)	

**Child risk taking behaviours**		OR (95% CI)		p	aOR (95% CI)	p	IRR (95% CI)	p	aIRR (95% CI)	p
**Measurement wave**				< 0.001		< 0.001		0.006		0.008
9-years		1 (reference)			1 (reference)					
11-years		1.16 (0.79, 1.69)			1.16 (0.79, 1.70)		1 (reference)		1 (reference)	
14-years		2.12 (1.47, 3.08)			2.19 (1.50, 3.19)		1.86 (1.19, 2.91)		1.83 (1.17, 2.88)	
**Highest education qualification at baseline**				0.76		0.42		0.073		0.39
No formal qual		1.18 (0.76, 1.83)			1.39 (0.85, 2.26)		1.50 (0.77, 2.94)		1.39 (0.65, 2.95)	
Secondary school		1.09 (0.69, 1.72)			1.20 (0.74, 1.96)		0.63 (0.24, 1.65)		0.81 (0.31, 2.13)	
Post-secondary school		1 (reference)			1 (reference)		1 (reference)		1 (reference)	
**Further study (over 0–6 years)**				0.71		0.92		0.017		0.013
Yes		1.08 (0.73, 1.59)			1.02 (0.68, 1.52)		0.24 (0.08, 0.78)		0.22 (0.07, 0.73)	
No		1 (reference)			1 (reference)		1 (reference)		1 (reference)	
**Student currently**				0.18		0.17				
Yes		1.51 (0.83, 2.74)			1.53 (0.83, 2.81)		unestimatable		unestimatable	
No		1 (reference)			1 (reference)					

**Child depression**		IRR (95% CI)		p	aIRR (95% CI)	p	IRR (95% CI)	p	aIRR (95% CI)	p
**Measurement wave**				< 0.001		< 0.001		< 0.001		< 0.001
9-years		1 (reference)			1 (reference)					
11-years		0.68 (0.63, 0.74)			0.68 (0.63, 0.74)		1 (reference)		1 (reference)	
14-years		0.53 (0.47, 0.59)			0.53 (0.47, 0.59)		0.78 (0.68, 0.89)		0.79 (0.69, 0.91)	
**Highest education qualification at baseline**				0.072		0.10		0.31		0.16
No formal qual		1.16 (1.01, 1.33)			1.14 (0.98, 1.33)		1.03 (0.82, 1.29)		1.05 (0.82, 1.35)	
Secondary school		1.16 (1.01, 1.34)			1.16 (1.01, 1.35)		0.89 (0.69, 1.15)		0.84 (0.65, 1.09)	
Post-secondary school		1 (reference)			1 (reference)		1 (reference)		1 (reference)	
**Further study (over 0–6 years)**				0.067		0.13		0.99		0.84
Yes		0.90 (0.80, 1.01)			0.91 (0.81, 1.03)		1.00 (0.78, 1.29)		1.03 (0.79, 1.33)	
No		1 (reference)			1 (reference)		1 (reference)		1 (reference)	
**Student currently**				0.31		0.26		0.21		0.24
Yes		1.15 (0.88, 1.51)			1.17 (0.89, 1.52)		0.61 (0.29, 1.32)		0.63 (0.29, 1.37)	
No		1 (reference)			1 (reference)		1 (reference)		1 (reference)	

Crude estimates controlled for measurement wave, highest education qualification at baseline, further study (over 0–6 years), and student currently variables; adjusted estimates controlled for measurement wave, highest education qualification at baseline, further study (over 0–6 years), student currently, age group, ethnicity, years lived in New Zealand, marital status, and cultural orientation. Additionally, for mothers, adjusted estimates were also controlled for household income and household size.

For analyses of children’s risk taking behaviours and mothers’ education, a significant increase by measurement wave was observed (p < 0.001). However, no difference in risk taking behaviours was observed for baseline highest education qualification (crude p = 0.76 and adjusted p = 0.42), further 0–6 years postpartum study (crude p = 0.71 and adjusted p = 0.92), or being a current student (crude p = 0.18 and adjusted p = 0.17). Among fathers, the low levels of current study forced this variable out of the model. After accounting for the significant measurement wave differences, fathers’ baseline highest education qualification was not associated with children’s risk taking behaviours (crude p = 0.073 and adjusted p = 0.39) but fathers who engaged in study over the 0–6 years postpartum period had children with significantly lower odds of risk taking behaviours at 11-years and 14-years measurement waves compared to 9-years levels than those who did not study (crude p = 0.017 and adjusted p = 0.013).

Finally, when considering child depression and mothers education, a significant decrease in CDI:S by measurement wave was observed (p < 0.001). No significant difference in CDI:S scores was observed for baseline highest education qualification (crude p = 0.072 and adjusted p = 0.10), further 0–6 years postpartum study (crude p = 0.067 and adjusted p = 0.13), or being a current student (crude p = 0.31 and adjusted p = 0.26). Among fathers, after accounting for the significant measurement wave differences, baseline highest education qualification was not associated with children’s CDI:S scores (crude p = 0.31 and adjusted p = 0.16), further 0–6 years postpartum study (crude p = 0.99 and adjusted p = 0.84), or being a current student (crude p = 0.21 and adjusted p = 0.24).

### Pattern of attrition over time

Using binomial GEE models to assess the pattern of attrition, the level of missing values significantly increased over time for mothers and fathers (both p < 0.001). Compared to baseline, mothers attrition was unrelated to ethnicity (p = 0.17), being a student (p = 0.87), years lived in New Zealand (p = 0.47), relationship status (p = 0.09), household income (p = 0.09), or family size (p = 0.24). However, significant missing data patterns emerged for: mothers’ age (p = 0.019), with older mothers less likely to attrite over time; mothers highest education qualification (p = 0.017), with those having no formal education more likely to attrite; and, cultural orientation (p = 0.046), with marginalists being more likely to attrite. Amongst father, attrition was unrelated to ethnicity (p = 0.12), highest education qualification (p = 0.22), being a student (p = 0.060), years lived in New Zealand (p = 0.96), or cultural orientation (p = 0.45). However, fathers’ age was associated with attrition (p = 0.014), with those aged 20–39 years at baseline more likely to attrite; as was relationship status (p = 0.002), with fathers who were single at baseline less likely with continued participation.

## Discussion

A novel and key finding identified here is that further study or acquisition of qualifications by parents within the 0–6 years postpartum period was associated with significantly lower childhood ln(BMI) increases at 11-years and 14-years measurement waves compared to 9-years levels than for children whose parents did not study. Should this relationship hold and be replicated elsewhere, then it has important implications. Addressing childhood obesity is a priority in New Zealand^36^, and Pacific children are disproportionately burdened through a multiplicity of social determinants of health yet evidence for efficacious interventions is lacking^37^. Of interest, and arguably adding strength to this identified relationship, was that the same direction and strength of this association was identified in the adjusted analyses undertaken separately for mothers and fathers. Conversely, baseline education levels of mothers or fathers were not associated with ln(BMI) in their children at ages 9–14 years in these adjusted analyses. Results from a 12 country study reveal a complex and varied relationship between parental education and body size, which appeared to be contingent on the developmental stage of different countries rather than parental education profiles^38^. Moreover, should different effects exist between prior educational levels, and current study engagement variables, in addition to important contextual and cultural factors, then these complex and varied relationships become even harder to disentangle.

As consistently observed elsewhere, mother’s increased baseline level of education was significantly associated with decreased CDI:S scores of their Pacific children in the late childhood and early adolescence period^39,40^. While not statistically significant, additionally and noteworthy was the large estimated effect size associated with further study or acquisition of qualifications for mothers within the 0–6 years postpartum period and lower CDI:S scores. If this estimated effect size holds, and is not simply a product of sampling variation, then it has important ramifications. Mental health illness is prevalent among today’s adolescents, and is an important public health issue accounting for 16% of the global burden of disease and injury in people aged 10–19 years^41^. Half of all mental health disorders in adulthood start by age 14 years, with most cases are undetected and untreated^42^. Again, Pacific people carry a higher burden of mental disorder than non-Pacific people^14,15^, yet are much less likely to access mental health services—especially among those aged under 20 years^43^. Therefore, identifying modifiable factors that may mitigate this burden is paramount.

Interestingly, the measured effect size was not seen between fathers’ education and their children’s mental health. Parental genetics explain a relative small variance in depression and anxiety symptoms in children^44^, suggesting a large nurture effect steeped in traditional children rearing practices, responsibilities and attachments; together with fathers having lower levels of nurturance and parental involvement, and lower levels of education or educational engagement^45^. Finding from Te Rau Hinengaro, a nationwide mental health survey of adults aged ≥ 16 years conducted between 2003 and 2004, showed that the prevalence of mental disorder declines in Pacific people monotonically with age of migration to New Zealand, which also suggests that genetic factors are not the primary driver of mental disorder^46^.

Another notable finding was the relationship between further study or acquisition of qualifications for fathers within the 0–6 years postpartum period and child risk taking behaviours in late childhood and early adolescence. Here, the estimate adjusted OR = 0.22 (95% confidence interval [CI]: 0.07, 0.73); again a large estimated effect size. The mechanism for this significant relationship in fathers (but not for mothers) requires further investigation, but may result from the positive childhood behavioural advantage associated with early and more engaged paternal involvement^47^. Parental monitoring—which may be an aspect of more-engaged parenting—has been found to be a protective factor against binge drinking, cigarette smoking, and suicidal behaviours amongst Pacific teenagers^48,49^.

Finally, in terms of the other time-varying education variables investigated, neither noteworthy nor significant associations between parental engagement and child outcomes were observed in this study over the 9-years to 14-years periods. This may simply reflect the relatively small statistical power to find differences, due to the small numbers, or that the parental education acquisition effect is more influential in children’s earlier years.

This study has a number of important salient strengths and limitations. It is a relatively large, diverse birth cohort that involves the mother-father-child triad, prospectively followed over time with multiple measurement waves and repeated assessments. Furthermore, the outcome variables are based on children’s own interviews and assessments, matched to self-reported maternal and paternal responses. Both BMI and CDI:S variables have robust psychometric properties. All three outcomes have considerable relevance to today’s adolescents. Moreover, a careful longitudinal statistical analysis was undertaken, with apposite likelihood functions and intra-participant correlation matrices employed. However, the childhood risk taking behaviour variable was created for this analysis, as was the variable measuring further study or acquisition of qualifications within the 0–6 years postpartum period. While having reasonable face validity, the psychometric properties of these two primary variables are unknown. The latter may also be subjected to misclassification bias, with mothers or fathers being students between, but not at, measurement waves and not being classified as such. Similarly, biases may results from parents being a student at a particular measurement wave but attriting or failing early-on in that study. This pragmatically defined relatively crude measure of further study or qualification acquisition for parents may have contributed to the small counts observed. Should this variable definition yield systematic misclassification, then biased estimates will likely result limiting the external validity of this study’s findings. Ideally, future research will adopt a more nuanced measure of ongoing parental education. Furthermore, while the three outcome variables were chosen for their priority within the New Zealand context, the choice was also made on pragmatic grounds (i.e., their availability over the 9-years, 11-years and 14-years waves) and other important health variables or domains might be considered. And while three pou were represented within these analyses, the fourth (spirituality) was neither listed as a priority area within the Ministry of Health Pacific health overview^19^ nor captured within the 9-years, 11-years and 14-years measurement instruments, despite its importance. Apart from religious affiliation, spirituality is rarely included in health or population surveys, yet deserves attention.

Participant composition varied over different measurement waves, and attrition since baseline was significantly related to mothers’ highest education qualification and other measured parental sociodemographic characteristics. A strength of GEE models is that if these missing data can be considered to be missing completely at random or missing at random, then robust estimates can be derived^50^. However, if these data are missing not at random, then bias estimates are likely to result. Thus, arguably, the largest threat to this study’s validity are these significant patterns of attrition together with unmeasured confounding effects. Unmeasured confounding variables can result in substantial bias in estimated exposure-outcome relationships, particularly if they are uncorrelated with the measured explanatory variables^51^. It might also be opined that the identified significant continued parental education variables found in these analyses may actually proxy other underlying unmeasured factors. Study replication using different suites of variables is needed to understand the impact of this unmeasured confounding phenomenon.

## Conclusions

Further maternal and paternal schooling during the six years after the birth of their child was identified as being differentially associated with various health indicators in their Pacific children during late childhood and early adolescence. It may be that those seeking additional study have an underlying intrinsic motivation or latent resource and capacity differences from those who did not study, resulting in better preventive parenting practices. If so the education variable used here may be a marker of unmeasured parental behaviours and practices, and family environments. Further research is needed to replicate these findings. Nonetheless, the potential personal, familial and policy impact is substantial—with individual and intergenerational consequences. These results align with and underscore the Ministry of Health’s Ola Manuia Pacific Health and Wellbeing Action Plan focusing on increasing educational opportunities for Pacific people as one mechanism for better, fairer and more equitable health outcomes.

## Supplementary Information

Supplementary Information.

## Data Availability

The datasets used for statistical analysis are held by the PIF Study directorate (see https://phrc.aut.ac.nz/our-research/pacific-islands-families-study). Application to use these data must be made through this directorate.
