# Supplementary material for: Parental education related to their children’s health in late childhood and early adolescence for Pacific families within New Zealand
Source: Sci Rep. 2022 Mar 29;12:5313. doi: 10.1038/s41598-022-09282-x (PMC8964731; doi:10.1038/s41598-022-09282-x)
Supplement: Supplementary file 1 — Supplementary Information. [file 41598_2022_9282_MOESM1_ESM.docx]

**Supplementary materials for:**

**Parental education related to their children’s health in late childhood and early adolescence for Pacific families within New Zealand**

Philip J. Schluter*^1,2^, Jesse Kokaua^3^, El-Shadan Tautolo^4^, Leon Iusitini^4^, Rosalina Richards^3^, Troy Ruhe^3^

^1^University of Canterbury – Te Whare Wānanga o Waitaha, School of Health Sciences – Te Kura Mātai Hauora, and Child Well-being Research Institute – Te Kāhui Pā Harakeke, Christchurch, New Zealand

^2^The University of Queensland, School of Clinical Medicine, Primary Care Clinical Unit, Brisbane, Australia

^3^University of Otago, Division of Health Sciences, Va’a o Tautai, Dunedin, New Zealand

^4^Auckland University of Technology, Centre for Pacific Health and Development Research, Auckland, New Zealand

**Corresponding author*:

Professor Philip Schluter, School of Health Sciences – Te Kura Mātai Hauora, University of Canterbury – Te Whare Wānanga o Waitaha, Private Bag 4800, Christchurch 8140, NEW ZEALAND.

E-mail: philip.schluter@canterbury.ac.nz; Tel: +64 3 366 7001.

**Figure S1.** Measurement waves and mother, father and child participant numbers included within the study.

|  | |  |  | |  |  | |  |  | |  |  | |  |  | |  | **Child**  **n=874** | |  | **Child**  **n=935** | |  | **Child**  **n=916** | |
| --- | --- | --- | --- | --- | --- | --- | --- | --- | --- | --- | --- | --- | --- | --- | --- | --- | --- | --- | --- | --- | --- | --- | --- | --- | --- |
|  |  |  |  |  |  |  |  |  |  |  |  |  |  |  |  |  |  |  |  |  |  |  |  |  |  |
|  |  |  |  |  |  |  |  |  |  |  |  |  |  |  |  |  |  |  |  |  |  |  |  |  |  |
|  |  |  |  |  |  | **Father**  **n=821** | |  | **Father**  **n=754** | |  |  |  |  | **Father**  **n=571** | |  |  |  |  | **Father**  **n=708** | |  | **Father**  **n=635** | |
|  |  |  |  |  |  |  |  |  |  |  |  |  |  |  |  |  |  |  |  |  |  |  |  |  |  |
|  |  |  |  |  |  |  |  |  |  |  |  |  |  |  |  |  |  |  |  |  |  |  |  |  |  |
| **Mother**  **n=1,477** | |  | **Mother**  **n=1,368** | |  | **Mother**  **n=1,201** | |  | **Mother**  **n=1,127** | |  | **Mother**  **n=1,024** | |  | **Mother**  **n=964** | |  | **Mother**  **n=909** | |  | **Mother**  **n=964** | |  | **Mother**  **n=886** | |
|  |  |  |  |  |  |  |  |  |  |  |  |  |  |  |  |  |  |  |  |  |  |  |  |  |  |
|  |  |  |  |  |  |  |  |  |  |  |  |  |  |  |  |  |  |  |  |  |  |  |  |  |  |
|  |  |  |  |  |  |  |  |  |  |  |  |  |  |  |  |  |  |  |  |  |  |  |  |  |  |
| **Birth**  **2000** | |  | **6-weeks**  **2000** | |  | **1-year**  **2001** | |  | **2-years**  **2002** | |  | **4-years**  **2004** | |  | **6-years**  **2006** | |  | **9-years**  **2009** | |  | **11-years**  **2011** | |  | **14-years**  **2014** | |
|  |  |  |  |  |  |  |  |  |  |  |  |  |  |  |  |  |  |  |  |  |  |  |  |  |  |

**Figure S2.** Empirical distribution of children’s logarithmically transformed body mass index (BMI) scores by measurement wave.

**Figure S3.** Empirical distribution of the 10-item Children’s Depression Inventory short form (CDI:S) scores by measurement wave.
